# Reconstructed spatial resolution and contrast recovery with Bayesian penalized likelihood reconstruction (Q.Clear) for FDG-PET compared to time-of-flight (TOF) with point spread function (PSF)

**DOI:** 10.1186/s40658-020-0270-y

**Published:** 2020-01-10

**Authors:** Julian M. Rogasch, Said Suleiman, Frank Hofheinz, Stephanie Bluemel, Mathias Lukas, Holger Amthauer, Christian Furth

**Affiliations:** 1 Charité–Universitätsmedizin Berlin, corporate member of Freie Universität Berlin, Humboldt-Universität zu Berlin, and Berlin Institute of Health, Department of Nuclear Medicine, Augustenburger Platz 1, Berlin, Germany; 2 Helmholtz-Zentrum Dresden-Rossendorf, Institute for Radiopharmaceutical Cancer Research, Dresden, Germany

**Keywords:** PET, Image reconstruction, Spatial resolution, Contrast recovery, Signal-to-noise ratio, TOF, PSF, Q.Clear, GE Discovery MI

## Abstract

**Background:**

Bayesian penalized likelihood reconstruction for PET (e.g., GE Q.Clear) aims at improving convergence of lesion activity while ensuring sufficient signal-to-noise ratio (SNR). This study evaluated reconstructed spatial resolution, maximum/peak contrast recovery (CRmax/CRpeak) and SNR of Q.Clear compared to time-of-flight (TOF) OSEM with and without point spread function (PSF) modeling.

**Methods:**

The NEMA IEC Body phantom was scanned five times (3 min scan duration, 30 min between scans, background, 1.5–3.9 kBq/ml F18) with a GE Discovery MI PET/CT (3-ring detector) with spheres filled with 8-, 4-, or 2-fold the background activity concentration (SBR 8:1, 4:1, 2:1). Reconstruction included Q.Clear (beta, 150/300/450), “PSF+TOF_4/16_” (iterations, 4; subsets, 16; in-plane filter, 2.0 mm), “OSEM+TOF_4/16_” (identical parameters), “PSF+TOF_2/17_” (2 it, 17 ss, 2.0 mm filter), “OSEM+TOF_2/17_” (identical), “PSF+TOF_4/8_” (4 it, 8 ss, 6.4 mm), and “OSEM+TOF_2/8_” (2 it, 8 ss, 6.4 mm). Spatial resolution was derived from 3D sphere activity profiles. RC as (sphere activity concentration [AC]/true AC). SNR as (background mean AC/background AC standard deviation).

**Results:**

Spatial resolution of Q.Clear_150_ was significantly better than all conventional algorithms at SBR 8:1 and 4:1 (Wilcoxon, each *p* < 0.05). At SBR 4:1 and 2:1, the spatial resolution of Q.Clear_300/450_ was similar or inferior to PSF+TOF_4/16_ and OSEM+TOF_4/16_. Small sphere CRpeak generally underestimated true AC, and it was similar for Q.Clear_150/300/450_ as with PSF+TOF_4/16_ or PSF+TOF_2/17_ (i.e., relative differences < 10%). Q.Clear provided similar or higher CRpeak as OSEM+TOF_4/16_ and OSEM+TOF_2/17_ resulting in a consistently better tradeoff between CRpeak and SNR with Q.Clear. Compared to PSF+TOF_4/8_/OSEM+TOF_2/8_, Q.Clear_150/300/450_ showed lower SNR but higher CRpeak.

**Conclusions:**

Q.Clear consistently improved reconstructed spatial resolution at high and medium SBR compared to PSF+TOF and OSEM+TOF, but only with beta = 150. However, this is at the cost of inferior SNR with Q.Clear_150_ compared to Q.Clear_300/450_ and PSF+TOF_4/16_/PSF+TOF_2/17_ while CRpeak for the small spheres did not improve considerably. This suggests that Q.Clear_300/450_ may be advantageous for the 3-ring detector configuration because the tradeoff between CR and SNR with Q.Clear_300/450_ was superior to PSF+TOF_4/16_, OSEM+TOF_4/16_, and OSEM+TOF_2/17_. However, it requires validation by systematic evaluation in patients at different activity and acquisition protocols.

## Background

The current clinical standard for image reconstruction in positron emission tomography (PET) is iterative algorithms, mainly ordered subset expectation maximization (OSEM). Recently developed systems commonly combine time-of-flight (TOF) capabilities and compensation for spatial variances in the scanner’s point spread function (PSF). While TOF mainly improves signal-to-noise ratio (SNR) at comparable convergence level [1] and leaves standardized uptake values (SUV) comparably unaffected [2, 3], PSF primarily increases reconstructed spatial resolution [4, 5] and has shown SUVmax increases by > 30% in clinical studies [2, 3]. Both TOF and PSF benefit the tradeoff between contrast recovery (CR) and SNR, but all conventional reconstruction methods share the principle limitation that adequate CR by increasing numbers of iterations/subsets will be at the cost of decreasing SNR.

Bayesian penalized likelihood reconstruction, such as GE Q.Clear, has been introduced to offer full convergence of focal activity peaks and high SNR in homogenous areas within the same PET dataset [6]. Q.Clear utilizes voxel-wise regulation of the iterative steps with a user-defined penalization factor β. Using a GE Discovery IQ scanner with analog detectors, Reynés-Llompart et al. showed an improved tradeoff between CR and SNR in a NEMA NU-2012 phantom protocol for Q.Clear with a β of 350 compared to PSF or OSEM (both without TOF) [7]. However, differences to conventional reconstruction methods are influenced by the choice of β. Lindström et al*.* reported superior contrast-to-noise ratios (CNR) in a NEMA IQ phantom for Q.Clear compared to OSEM with PSF and TOF for a β of 400 and 533 but inferior CNR for a β < 300 [8]. While the improved CR at a β = 150 can result in higher SUVmax in small pulmonary nodules by 25% [9], such significant intermethod SUV differences may not be observed at β values > 150 [10] and may only be achieved at the cost of high image noise [8]. Furthermore, the performance of TOF and PSF is dependent on the signal-to-background ratio (SBR) and lesion/sphere size [11, 12]. Previous studies that compared Q.Clear and conventional methods by only evaluating isolated reconstruction settings may therefore be of limited representativeness.

The aim of the current study was to generate a differentiated insight into performance benefits provided by the Q.Clear algorithm as measured by reconstructed spatial resolution, CR and SNR. This should facilitate the identification of the most appropriate algorithm for clinical use with the 3-ring digital detector configuration of the GE Discovery MI. Different β settings in Q.Clear were compared to different iterations/subsets and filter settings for OSEM with TOF with or without PSF.

## Methods

### Phantom imaging

Three phantom measurement series were performed using the GE Discovery MI PET scanner (GE Healthcare, General Electric, Boston, MA, USA) with a digital 3-ring detector and a reported sensitivity of 7.3 cps/kBq [13]. Total activity in the field of view was approximately 35 MBq. The absolute activities were measured in the same dose calibrator that is used for periodic calibration of the PET system (ISOMED 2010, MED Dresden GmbH, Germany). To achieve SBR of 2:1, 4:1, and 8:1, the six spheres of a NEMA IEC PET Body Phantom (manufacturer, PTW Freiburg, Germany; sphere diameter, 10, 13, 17, 22, 28, and 37 mm) were filled with 7.5, 12.5, or 24.4 kBq/ml 18F-fluoride, respectively (decay-corrected to the start of the first imaging). The background was filled with either 3.9, 3.1, or 3.1 kBq/ml (true SBR, 1.94:1, 4.06:1, and 7.87:1). Imaging of each phantom was performed for five times every 30 min for 3 min each with the sphere plane aligned to the isocenter of the single bed position. CT data of the phantom obtained for each scan were used for attenuation and scatter correction.

### Image reconstruction

PET raw data were reconstructed using Q.Clear with a β value of 150, 300, or 450, respectively (Q.Clear_150_, Q.Clear_300_, Q.Clear_450_). OSEM+TOF reconstruction (GE VUE Point FX) was performed as OSEM+TOF_4/16_ (4 iterations, 16 subsets, Gaussian in-plane filter of 2.0 mm), OSEM+TOF_2/17_ (2 iterations, 17 subsets, 2.0 mm filter), and OSEM+TOF_2/8_ (2 it, 8 ss; 6.4 mm). OSEM+PSF+TOF (hereafter referred to as PSF+TOF; GE VUE Point FX with SharpIR) was reconstructed as PSF+TOF_4/16_ (4 it, 16 ss, 2.0 mm), as PSF+TOF_2/17_ (2 it, 17 ss, 2.0 mm) and PSF+TOF_4/8_ (4 it, 8 ss, 6.4 mm). OSEM+TOF and PSF+TOF reconstructions always included a “standard” *z*-axis filter. In all 9 reconstruction settings, field of view was 70 cm (matrix size, 256 × 256; voxel size, 2.73 × 2.73 × 2.78 mm^3^).

### Image assessment

Measurement of activity concentrations (AC; kBq/ml) was performed with dedicated software (ROVER, version 3.0.34, ABX advanced biochemical compounds GmbH, Radeberg, Germany). The mean background AC and its standard deviation (SD) were defined with a spherical background volume of interest (VOI; volume, 30 ml). Maximum AC of each sphere was obtained. Using the ACCURATE tool (version v23102018, Ronald Boellaard, Amsterdam UMC, Amsterdam, The Netherlands), the peak AC of each sphere was defined as the average AC in a spherical VOI of 1.2 cm in diameter that was positioned to generate the highest peak AC of this sphere. Peak and maximum CR (CRpeak, CRmax) were defined as the ratio of measured peak/maximum AC of the spheres to the true decay-corrected AC.

SNR was defined as follows:

$$ \mathrm{SNR}=\frac{\ \mathrm{background}\ \mathrm{mean}\ \mathrm{AC}}{\mathrm{background}\ \mathrm{AC}\ \mathrm{standard}\ \mathrm{deviation}} $$

**Fig. 1:**
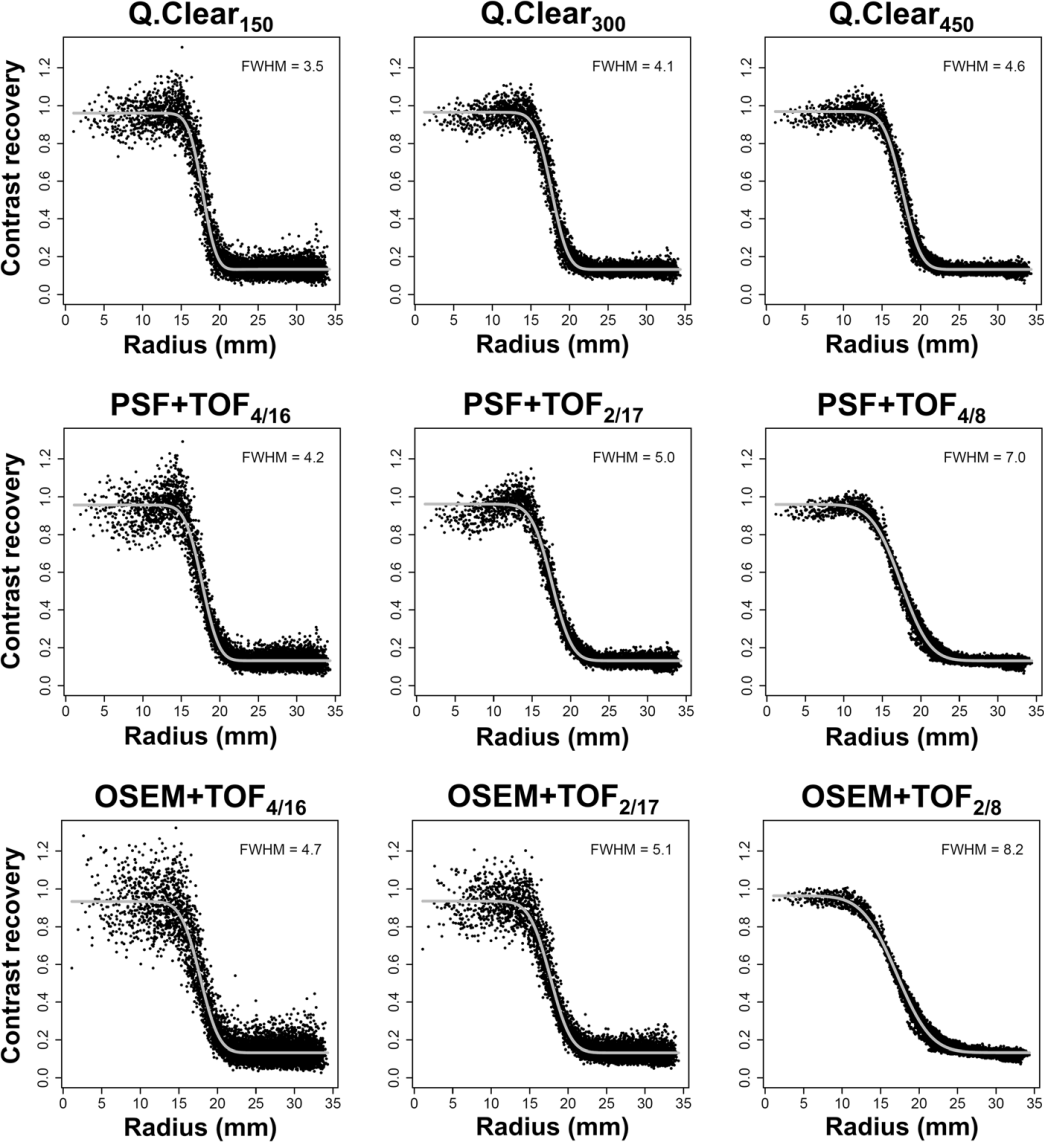
Sphere activity profiles (37 mm). Radial activity profiles of the 37 mm sphere are displayed for signal-to-background ratio (SBR) 8:1 with the respective reconstructed spatial resolution (full width at half maximum, FWHM)

**Fig. 2:**
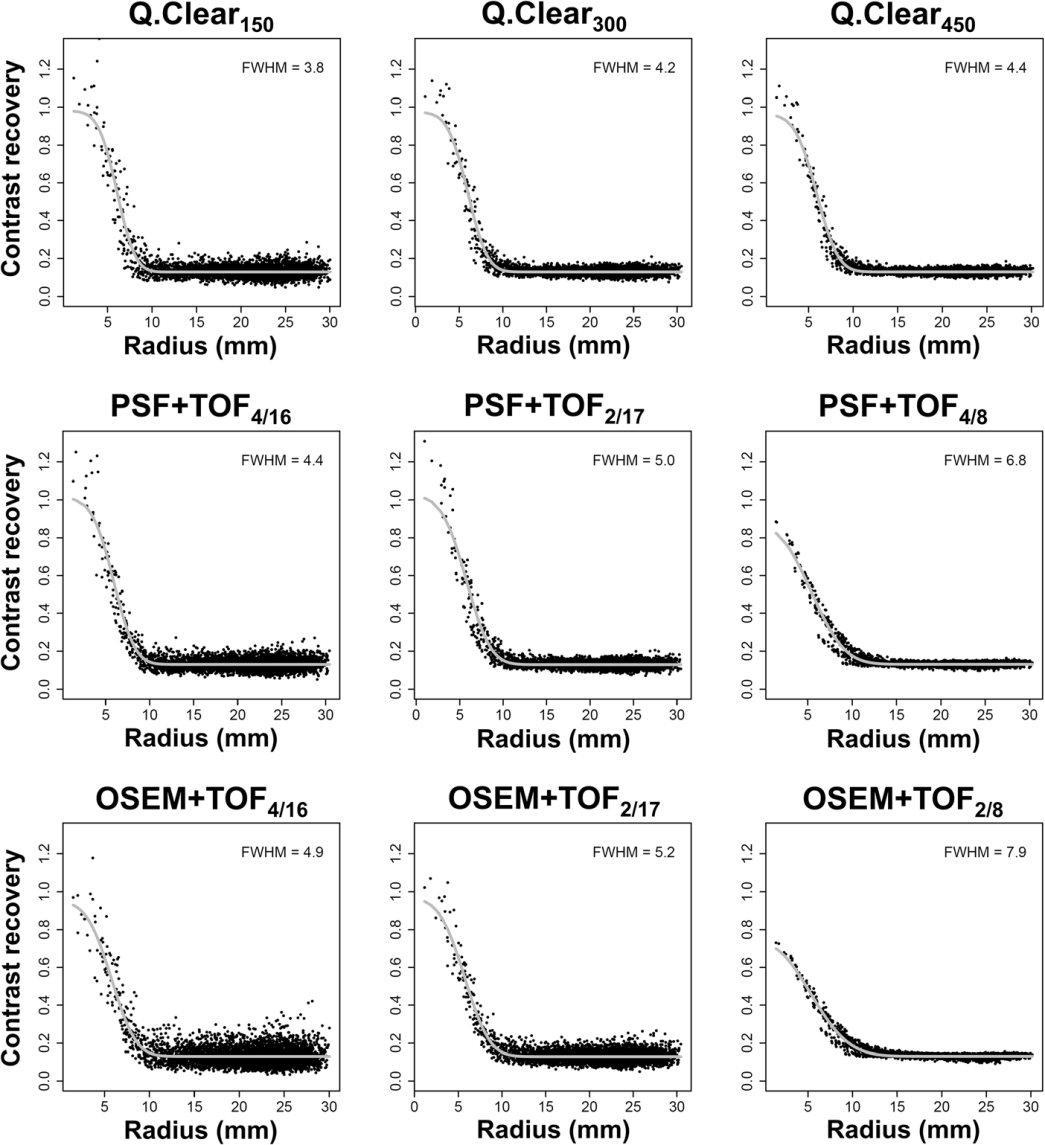
Sphere activity profiles (13 mm). Radial activity profiles of the 13 mm sphere are displayed for signal-to-background ratio (SBR) 8:1 with the respective reconstructed spatial resolution (full width at half maximum, FWHM)

The spatial resolution was assessed as the full width at half maximum (FWHM) of the PSF in the reconstructed images. PSF was modeled by a 3D Gaussian, and FWHM was determined by applying the method described in detail by Hofheinz et al. [14]. This method is based on fitting the analytic solution for the radial activity profile of a homogeneous sphere convolved with a 3D Gaussian to the reconstructed data. In this process, the full 3D vicinity of each sphere is evaluated by transforming the data to spherical coordinates relative to the respective sphere's center (Fig. 1 and Fig. 2). The analytic solution has five parameters: signal (true activity within the sphere), background level, FWHM of the PSF, the sphere radius, and the wall thickness of the spherical inserts. Sphere radius and wall thickness were fixed to their known values. The remaining three parameters were determined by non-linear least-squares fits. With this method the spatial resolution can be determined at finite background as well as for extended objects, and, therefore, allows to study size and contrast dependence of the resolution. Note that this method assumes a Gaussian PSF, which is never exactly the case. However, the method still leads to a reasonable approximation of the spatial resolution as long as the slope at the object boundary (signal decline) is modeled correctly by the fit function.

Inner volumes (25.6, 10.7, 5.4, 2.6, 1.2, and 0.58 ml), inner radii (18.3, 13.7, 10.9, 8.5, 6.6, and 5.2 mm), and wall thickness (range, 0.9 to 1.3 mm) of the spheres were determined based on weighting of the sphere inserts after filling with water (corrected for the volume of the tube mounting) combined with caliper measurements of the outer diameters.

### Patient example

**Fig. 3:**
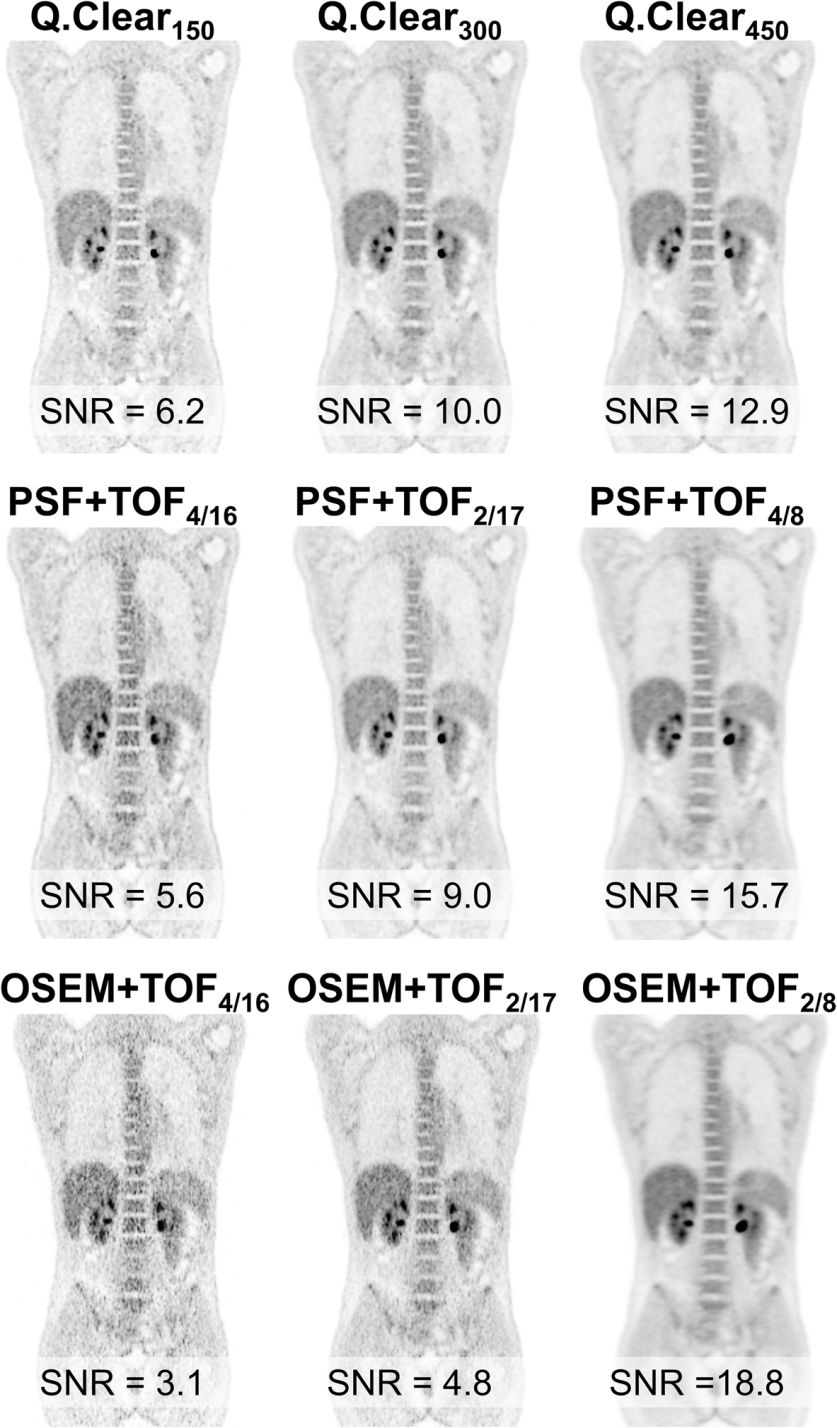
Patient example. Coronar FDG-PET images of a patient are displayed for all nine reconstruction algorithms (details see the “Methods” section). Signal-to-noise ratio (SNR) in the liver is given. Notably, Q.Clear_150_, PSF+TOF_4/16_, OSEM+TOF_4/16_, and OSEM+TOF_2/17_ exhibit unacceptably high noise (i.e., low SNR)

A patient example (Fig. 3) was included by way of illustration depicting a male patient (body weight, 63 kg; body mass index [BMI], 21.8 kg/m^2^) who underwent FDG-PET/CT for early response assessment for Hodgkin’s lymphoma using the same PET scanner. Injection of 250 MBq 18F-FDG was followed by acquisition in the supine position from base of the skull to proximal femora 62 min post-injection (decay-corrected injected activity at scan start, 2.7 MBq/kg; acquisition time, 3 min per bed position). Low-dose-CT was acquired for attenuation correction. PET raw data were reconstructed with the 9 reconstruction algorithms detailed above. SNR in the liver for each algorithm was calculated as the ratio of SUVmean to SUV standard deviation in a spherical VOI in the right liver lobe with an identical location in each dataset (volume, 19.2 ml).

### Statistical analysis

Statistical analysis was performed using SPSS 22 (IBM Corporation, Armonk, NY, USA). Descriptive parameters were expressed as mean and SD. Differences in spatial resolution, CRpeak, CRmax, and SNR between reconstruction methods were compared using the Wilcoxon test. Differences in these measures between SBR were compared with either Kruskal-Wallis test or Mann-Whitney *U* test. Statistical significance was generally assumed at *p* < 0.05.

## Results

### Reconstructed spatial resolution

Reconstructed spatial resolution was estimated based on an analytic fit to the spheres’ activity profiles which are depicted in Figs. 1 and 2 for the first imaging time point at SBR 8:1. These profiles demonstrate that only OSEM+TOF_2/8_ gives a smooth sphere profile but fails to reach high CR in the small sphere. In the large sphere (Fig. 1), overshoots at the sphere’s surface (edge artifacts) can be observed in similar shape for Q.Clear and PSF+TOF but with varying scatter of single voxels depending on the reconstruction parameters. Edge elevations with Q.Clear increase with decreasing β. At Q.Clear_300_, their magnitude is comparable to PSF+TOF_2/17_. At Q.Clear_150_, CR overshoot is more comparable to PSF+TOF_4/16_ and OSEM+TOF_2/17_. For PSF+TOF, they increase with increasing iterations/subsets and narrow in-plane filter (2.0 mm). OSEM+TOF_4/16_ and OSEM+TOF_2/17_ generally exceed a CR of 1.0 for the large sphere irrespective of the location along the sphere’s radius (primarily from excessive noise). In the small sphere (Fig. 2), overshoots are also visible, but due to the small radius, the typical shape of the edge elevations is not visualized.

For all nine reconstruction methods, spatial resolution (mean of all five scan time points) significantly decreased from SBR 8:1 to SBR 4:1 (Mann-Whitney *U* test, each *p* < 0.01) and from SBR 4:1 to SBR 2:1 (each *p* < 0.05; except for PSF+TOF_4/16_, PSF+TOF_2/17_, OSEM+TOF_2/17_, and PSF+TOF_4/8_, *p* > 0.05). This decline was especially observed for the Q.Clear algorithms (Table 1).

**Table 1:** Reconstructed spatial resolution

	Mean ± SD		
SBR 8:1			
Q.Clear_150_	3.7 ± 0.2		
Q.Clear_300_	4.3 ± 0.2		
Q.Clear_450_	4.7 ± 0.2		
PSF+TOF_4/16_		4.2 ± 0.2	
PSF+TOF_2/17_		5.1 ± 0.2	
PSF+TOF_4/8_		7.0 ± 0.2	
OSEM+TOF_4/16_			4.8 ± 0.2
OSEM+TOF_2/17_			5.2 ± 0.3
OSEM+TOF_2/8_			8.2 ± 0.2
SBR 4:1			
Q.Clear_150_	4.4 ± 0.6		
Q.Clear_300_	5.0 ± 0.5		
Q.Clear_450_	5.6 ± 0.4		
PSF+TOF_4/16_		4.7 ± 0.2	
PSF+TOF_2/17_		5.7 ± 0.6	
PSF+TOF_4/8_		7.5 ± 0.4	
OSEM+TOF_4/16_			5.1 ± 0.3
OSEM+TOF_2/17_			5.8 ± 0.7
OSEM+TOF_2/8_			8.8 ± 0.3
SBR 2:1			
Q.Clear_150_	5.1 ± 0.7		
Q.Clear_300_	5.9 ± 0.8		
Q.Clear_450_	6.4 ± 0.6		
PSF+TOF_4/16_		5.1 ± 0.5	
PSF+TOF_2/17_		6.2 ± 0.7	
PSF+TOF_4/8_		7.6 ± 0.5	
OSEM+TOF_4/16_			5.4 ± 0.7
OSEM+TOF_2/17_			6.0 ± 0.9
OSEM+TOF_2/8_			9.2 ± 0.6

Mean ± SD of the reconstructed spatial resolution (FWHM) for the three largest spheres is displayed (i.e., diameter of 22, 28, and 37 mm). Increasing SD mirror increasing noise with decreasing SBR

At SBR 8:1 and 4:1, Q.Clear_150_ yielded significantly better spatial resolution than all conventional algorithms (Wilcoxon test, each *p* < 0.05) while at SBR 2:1 spatial resolution was similar to PSF+TOF_4/16_ and OSEM+TOF_4/16_ (each *p* > 0.05). Q.Clear_300_ also showed significantly superior spatial resolution at SBR 8:1 compared to all conventional algorithms (each *p* < 0.05) except for PSF+TOF_4/16_ (*p* > 0.05). Spatial resolution for Q.Clear_300_ at SBR 4:1 and 2:1 was inferior to PSF+TOF_4/16_ (*p* < 0.05) and similar to OSEM+TOF_4/16_ (*p* > 0.05). Q.Clear_450_ was inferior to PSF+TOF_4/16_ (*p* < 0.05) and comparable to OSEM+TOF_4/16_ (*p* > 0.05) at SBR 8:1 while it was inferior to both PSF+TOF_4/16_ and OSEM+TOF_4/16_ at SBR 4:1 and 2:1 (each *p* < 0.05).

### SNR

Mean SNR significantly increased from Q.Clear_150_ to Q.Clear_300_ and Q.Clear_450_ (each *p* < 0.001; Table 2). Compared to conventional algorithms, SNR of Q.Clear_150_ was most similar to OSEM+TOF_2/17_ (*p* = 0.14) and PSF+TOF_4/16_ (*p* = 0.04) while Q.Clear_300_ showed similar SNR as PSF+TOF_2/17_ (*p* = 0.08). SNR of Q.Clear_450_ was significantly higher than of PSF+TOF_4/16_/PSF+TOF_2/17_ and OSEM+TOF_4/16_/OSEM+TOF_2/17_ (each *p* < 0.01). Figure 3 gives a patient example.

**Table 2:** SNR

	Mean ± SD		
Q.Clear_150_	4.2 ± 1.4		
Q.Clear_300_	7.0 ± 2.1		
Q.Clear_450_	9.3 ± 2.7		
PSF+TOF_4/16_		4.4 ± 1.1	
PSF+TOF_2/17_		7.0 ± 1.8	
PSF+TOF_4/8_		12.8 ± 3.2	
OSEM+TOF_4/16_			2.5 ± 0.6
OSEM+TOF_2/17_			3.9 ± 0.9
OSEM+TOF_2/8_			16.6 ± 4.1

Mean ± SD of the SNR is displayed (combined data of all three SBR)

### CRpeak (10, 13, and 17 mm spheres)

**Fig. 4:**
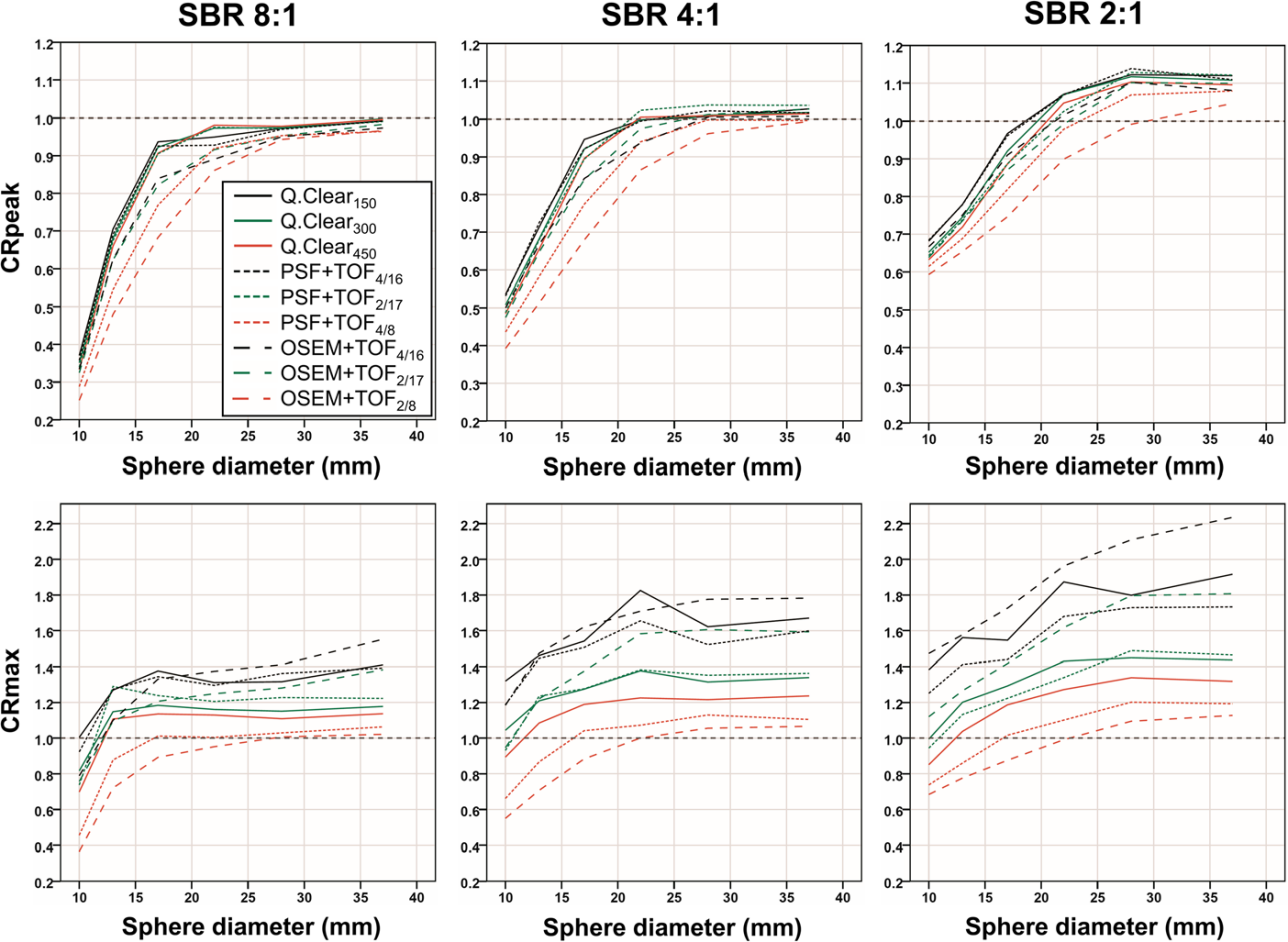
CRpeak and CRmax. Peak contrast recovery (CRpeak; upper row) and maximum contrast recovery (CRmax; lower row) are displayed for all reconstruction algorithms at the three signal-to-background ratios (SBR) as a function of sphere diameters

Relative CRpeak differences between Q.Clear_150_ and neither Q.Clear_300_ nor Q.Clear_450_ exceeded 10% at any SBR or sphere size (Fig. 4).

None of the differences between any Q.Clear reconstruction and PSF+TOF_4/16_ or PSF+TOF_2/17_ exceeded 10% at any SBR or sphere size, and intermethod differences did not differ between SBR (Kruskal-Wallis test, each *p* > 0.05). Relative CRpeak differences between Q.Clear_150_ or Q.Clear_300_ and either OSEM+TOF_4/16_, OSEM+TOF_2/17_, PSF+TOF_4/8_, or OSEM+TOF_2/8_ exceeded 10% in at least one of the three small spheres at all SBR, and differences increased with SBR (each *p* < 0.01). Relative differences between Q.Clear_450_ and OSEM+TOF_4/16_ and OSEM+TOF_2/17_ increased with SBR (*p* < 0.05) but did not surpass 10%. Differences in Q.Clear_450_ and PSF+TOF_4/8_ increased with SBR (*p* < 0.001) and exceeded 10% at SBR 8:1/4:1; differences to OSEM+TOF_2/8_ surpassed 10% at all SBR.

### Contrasting CRpeak differences with SNR differences

**Fig. 5:**
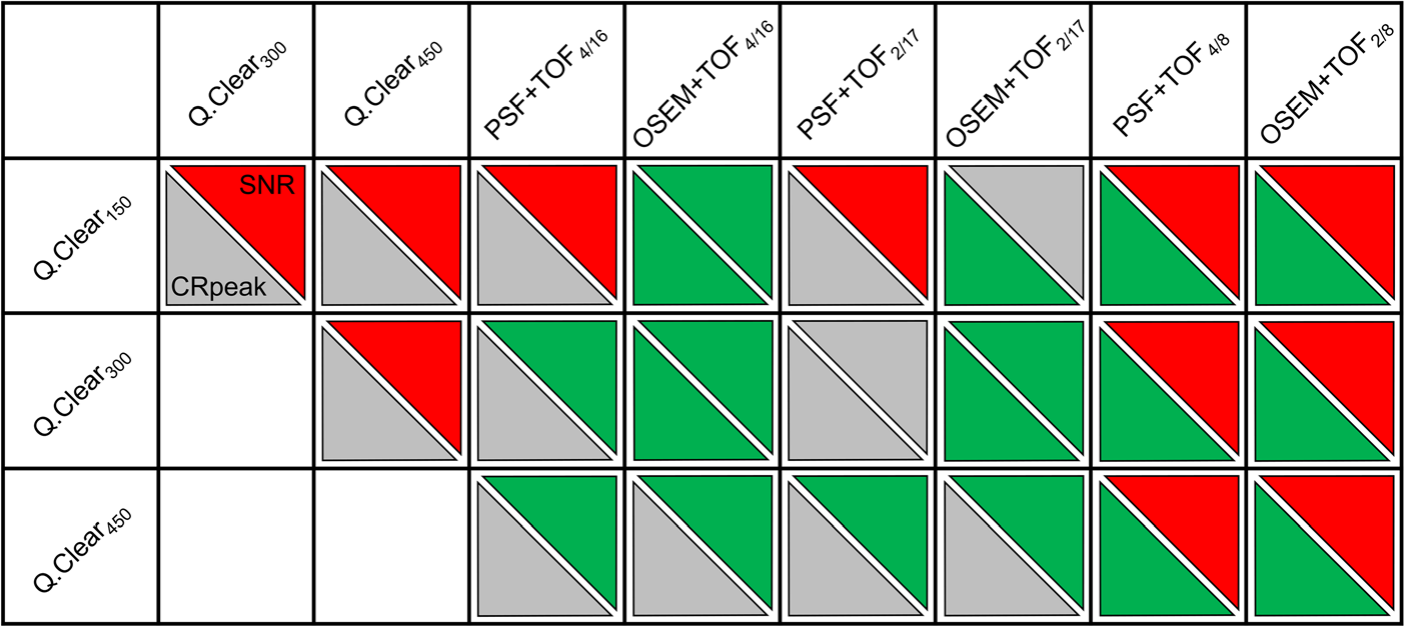
Contrasting CRpeak differences to SNR differences. Tradeoff between intermethod differences in peak contrast recovery (CRpeak) and signal-to-noise ratio (SNR) is displayed for different pairs of reconstruction algorithms as a combined analysis of all SBR. Colors of the triangles represent either higher (green), equal (grey) or lower (red) CRpeak or SNR for the algorithm in the left column, respectively. For example, if in a certain pair of algorithms, one provides higher CRpeak for the small spheres at similar SNR (i.e., green and grey triangle) or vice versa, this algorithm may be attested superior overall image quality. For CRpeak, higher or lower values are defined as > 10% difference for at least one of the smaller spheres (10, 13, and 17 mm). For SNR, statistically significant differences (Wilcoxon test) are determining

Figure 5 contrasts intermethod differences in CRpeak (three small spheres) and SNR of Q.Clear and conventional reconstruction methods. At similar SNR (mean relative difference of all SBR, + 6.3 ± 10.8%), Q.Clear_150_ showed higher CRpeak for the small spheres than OSEM+TOF_2/17_ (+ 10.6 ± 3.0%) while CRpeak was similar to PSF+TOF_4/16_ (+ 1.0 ± 2.8%) and PSF+TOF_2/17_ (+ 6.0 ± 2.6%) at lower SNR (PSF+TOF_4/16_, − 7.4 ± 8.0%; PSF+TOF_2/17_, − 41.4 ± 5.2%). Q.Clear_300_ resulted in both higher CRpeak and SNR than OSEM+TOF_2/17_ (CRpeak, + 6.9 ± 3.5%; SNR, + 78.0 ± 13.3%). CRpeak of Q.Clear_300_ and PSF+TOF_2/17_ were similar (+ 2.1 ± 2.2%) as was SNR (− 1.8 ± 5.9%). Q.Clear_450_ showed equal CRpeak as PSF+TOF_4/16_ (− 6.9 ± 3.9%) and PSF+TOF_2/17_ (− 1.5 ± 2.4%) at higher SNR (+ 107 ± 11.0%; + 31.1 ± 6.3%). CRpeak were also similar to OSEM+TOF_4/16_ (+ 0.1 ± 5.3%) and OSEM+TOF_2/17_ (+ 3.5 ± 4.0%) at higher SNR (+ 264 ± 26.1%; + 138 ± 15.0%). Compared to PSF+TOF_4/8_ and OSEM+TOF_2/8_, all Q.Clear methods showed higher CRpeak (Q.Clear_150_ vs. OSEM+TOF_2/8_, + 25.5 ± 6.4%) at lower SNR (Q.Clear_150_ vs. OSEM+TOF_2/8_, − 75.1 ± 2.5%).

### CRmax (10, 13, and 17 mm spheres)

CRmax of Q.Clear_150_ exceeded all other reconstruction methods by ≥ 10% for at least one of the three small spheres at all SBR (Fig. 4). Q.Clear_300_ showed comparable CRmax for the small spheres as PSF+TOF_4/16_ but surpassed CRmax of all other conventional algorithms by > 10%. Q.Clear_450_ showed lower CRmax (≥ 10%) than PSF+TOF_4/16_, PSF+TOF_2/17_, OSEM+TOF_4/16_, and OSEM+TOF_2/17_ but higher CRmax compared to PSF+TOF_4/8_ and OSEM+TOF_2/8_.

## Discussion

The present study assessed reconstructed spatial resolution, CR, and SNR in phantom measurements for Q.Clear in comparison to PSF+TOF and OSEM+TOF as a basis for investigations on the most appropriate algorithm for clinical use with the 3-ring-detector GE Discovery MI.

In general, differences between Q.Clear and conventional algorithms were considerably higher when using a lower product of iterations and subsets for PSF+TOF or OSEM+TOF (16 or 32 vs. 34 vs. 64) which is less likely to reach convergence and, especially, using a smoother in-plane postprocessing filter (6.4 mm vs. 2.0 mm). Using these parameters, PSF+TOF and OSEM+TOF consistently provided significantly inferior reconstructed spatial resolution than Q.Clear as well as lower CRpeak in the small spheres (diameter, 10 to 17 mm). In contrast, SNR were significantly higher than with any Q.Clear algorithm. With a high product of iterations and subsets and a 2.0 mm in-plane filter (PSF+TOF_4/16_ and OSEM+TOF_4/16_), CRpeak was similar to all Q.Clear algorithms, and SNR was comparable or even lower than with Q.Clear_150_. Both observations are in line with Teoh et al. who showed superior CR but lower SNR for Q.Clear with β of 100 to 400 compared to OSEM+TOF with 2 iterations, 24 subsets, and 6.4 mm filter (GE Discovery 690). However, compared to PSF+TOF with 3 iterations, 24 subsets, and 2.0 mm filter, CR and SNR with Q.Clear were either similar (β of 100 or 200) or progressively lower (β ≥ 300) [9]. For clinical use, the smooth appearance of images reconstructed with PSF+TOF_4/8_/OSEM+TOF_2/8_ may be appealing to physicians (Fig. 3), but it will result in a potential loss of small sphere detectability and CR.

However, the observation of higher CR at systematically lower SNR is of limited relevance when assessing actual superiority or inferiority in overall image quality, i.e., at the tradeoff between both measures. It remains a mere observation; therefore, pairs of reconstruction settings with comparable SNR were evaluated additionally. PSF+TOF_2/17_ showed similar SNR as Q.Clear_300_ while SNR in OSEM+TOF_2/17_ was comparable to Q.Clear_150_. Comparison of Q.Clear_300_ and PSF+TOF_2/17_ showed that despite superior reconstructed spatial resolution for Q.Clear_300_ at SBR 8:1 and 4:1 (4.3/5.0 mm vs. 5.1/5.7 mm), both methods achieved similar CRpeak in the small spheres (i.e., relative differences ≤ 10%; Fig. 5). Comparing Q.Clear_150_ to OSEM+TOF_2/17_, both spatial resolution (3.7/4.4 mm vs. 5.2/5.8 mm) and CRpeak were higher for Q.Clear_150_, i.e., it offered superior image quality (tradeoff between CR and SNR). Image quality with the Q.Clear algorithms was generally superior to OSEM+TOF_4/16_ and OSEM+TOF_2/17_ but only Q.Clear_450_ was consistently superior to both PSF+TOF_4/16_ and PSF+TOF_2/17_ (Fig. 5). Vandendriessche et al. recently investigated the same PET scanner (also the 3-ring configuration) under the NEMA NU2-2012 protocol and obtained superior image quality (CR and background variability) for Q.Clear with β of 50 compared to OSEM+TOF with 4 iterations and 34 subsets (filter not detailed) [13]. Reynés-Llompart et al. also reported an improved tradeoff between CR and SNR for Q.Clear in measurements based on the NEMA NU2-2012 standard (Q.Clear with β of 350; OSEM and PSF with 4 iterations, 12 subsets, and 4.8 mm in-plane filter). CR at SBR 8:1, 4:1, and 2:1 was comparable or slightly higher with Q.Clear while background variability was generally lower [7]. However, the authors provided additional data on PSF and OSEM with 8 iterations, 12 subsets and an in-plane filter of 2.0 mm which revealed superior CR for OSEM and PSF compared to Q.Clear with β of 350 (Supplemental Data in [7]). Similar to the present study, this underlines the necessity of a differentiated comparison as for all Q.Clear algorithms currently investigated a PSF+TOF reconstruction with relatively similar image properties could be identified. Discrepancies between Reynés-Llompart et al. and the current measurements may arise from the different PET scanner (GE Discovery IQ) with analog detectors and considerably higher system sensitivity (21.6 vs. 7.3 cps/kBq) due to 5 rows of detector rings. Notably, background variability for the GE Discovery MI at the NEMA NU2-2012 standard has shown to decrease considerably when comparing the 3-ring to the 4-ring configuration while CR did not improve [13].

Radial activity profiles of the spheres were derived visualizing the cause of intermethod CR differences. In PSF reconstruction, the so-called edge artifacts are well documented as the cause of increased CR [11, 15]. As Q.Clear reconstruction also utilizes PSF compensation [6], Fig. 1 demonstrates edge artifacts which are similar to PSF+TOF and which increase with decreasing β [16] but are clearly detectable even at β = 450. The sphere profiles further illustrate that in the small sphere, maximum CR is determined by single voxels that may far surpass 1.0 and explain exceedingly high CRmax especially with Q.Clear_150_ and OSEM+TOF_4/16_. As a result, CRmax tends to overestimate the true sphere activity concentration even in the smaller spheres (Fig. 4). In contrast, activity distribution along the slope of the sphere profile appears similar for all Q.Clear methods and PSF+TOF_4/16_ and PSF+TOF_2/17_ which explains comparable CRpeak (Fig. 5).

The current analysis demonstrated superior reconstructed spatial resolution for Q.Clear compared to conventional algorithms at high SBR (8:1). Improving spatial resolution with increasing SBR can be seen in all reconstruction methods (Table 1) but especially in the three Q.Clear algorithms (each about 27% improvement for SBR 8:1 vs. 2:1). Conversely, spatial resolution at SBR 4:1 and 2:1 with PSF+TOF_4/16_ and OSEM+TOF_4/16_ was similar or even superior to Q.Clear_300_ and Q.Clear_450_. Although recent data on the spatial resolution for this PET scanner is available [13], it had been obtained based on the NEMA NU2-2012 protocol for a filled tube of ≤ 1 mm diameter, and comparative data for Q.Clear is generally not available. Previous reports suggest that spatial resolution (NEMA protocol) for the GE Discovery MI does not improve with an increasing number of detector rings [13, 17].

The phantom measurements were performed as representative as possible for imaging protocols in clinical routine, i.e., typical activity concentrations in spheres and background were chosen. In addition, the effective reconstructed spatial resolution was determined using spheres with typical sizes of tumor lesions and SBR. As illustrated by the patient example in Fig. 3, SNR of Q.Clear_150_ and PSF+TOF or OSEM+TOF with high products of iterations and subsets combined with a 2.0-mm in-plane filter may be insufficient for clinical use with the 3-ring detector configuration because high image noise could impair reliable lesion detection. Under these conditions, with sufficiently long acquisition time (3 min per bed position in the current study), Q.Clear_300_ and Q.Clear_450_ may offer the best compromise between CR and SNR for whole-body imaging. Lindström et al. used the 4-ring detector GE Discovery MI for phantom and patient examinations. The authors reported superior SNR at matched noise in a liver VOI with Q.Clear (β of 133 to 533) compared to PSF+TOF (3 it, 16 ss, 5.0 mm filter), and clinical whole-body FDG-PET data with β of 267 to 533 achieved the highest score in subjective image quality [8]. Trägårdh et al. found a β of 500 to 600 to be optimal for whole-body FDG-PET with the 4-ring detector GE Discovery MI if the product “AT” of injected activity per kilogram and acquisition time per bed position was 6 (i.e., 4 MBq/kg and 1.5 min acquisition) [18]. The same group recommended a β of 400 to 550 for 18F-fluorocholine PET if “AT” was 6 [19]. If “AT” is increased, this could enable lower β values such as 300 while maintaining sufficient SNR for clinical reporting. However, systematic data on the 3-ring detector configuration is currently missing, and the current patient example (Fig. 3; “AT” = 12) can only be illustrative. Dedicated investigations of patient data are necessary to confirm the most appropriate Q.Clear reconstruction setting at different BMI, levels of injected activity and acquisition times. Furthermore, the selection of the most suitable reconstruction setting may also differ depending on the radionuclide or the examined body part and clinical question (whole-body vs. brain studies).

## Conclusions

Q.Clear can provide superior reconstructed spatial resolution compared to PSF+TOF and OSEM+TOF if β is low (150) and SBR is high. However, using the 3-ring detector configuration, this is at the cost of inferior SNR with Q.Clear_150_ compared to Q.Clear_300/450_ and PSF+TOF_4/16_/PSF+TOF_2/17_ while CRpeak for the small spheres did not improve considerably. In contrast, Q.Clear_300/450_ showed an improved tradeoff between CR and SNR compared to PSF+TOF and OSEM+TOF with different combinations of iterations, subsets and in-plane filters. Especially compared to Q.Clear_150_, PSF+TOF_4/16,_ and OSEM+TOF_4/16_, this may allow for an appropriately low level of image noise for whole-body PET in clinical routine while averting disadvantageous small lesion detectability and quantification. However, a dedicated systematic evaluation of patient data is required for validation at different activity and acquisition protocols.

## Data Availability

The datasets generated and/or analyzed during the current study are available in the Zenodo repository, 10.5281/zenodo.3373973

## Abbreviations

AC: Activity concentration
BMI: Body mass index
CNR: Contrast-to-noise ratio
CR: Contrast recovery
FDG: Fluorodeoxyglucose
FWHM: Full width at half maximum
OSEM: Ordered subset expectation maximization
PET: Positron emission tomography
PSF: Point spread function
CRpeak/CRmax: Peak/maximum contrast recovery
SBR: Signal-to-background ratio
SD: Standard deviation
SNR: Signal-to-noise ratio
SUV: Standardized uptake value
TOF: Time-of-flight
VOI: Volume of interest
